# Estimating the cost of and willingness to pay for providing the dapivirine ring for HIV prevention in Kenya

**DOI:** 10.1186/s12889-025-22291-5

**Published:** 2025-03-22

**Authors:** Peter Stegman, Steven Forsythe, Urbanus Kioko, Millicent Kiruki, Patriciah Jeckonia, Martha Larson, Delivette Castor, Guy Mahiane, Maryline Mireku, Mike Ekisa, Kristine Torjesen, Mary Mugambi, Daniel Were, Katharine Kripke

**Affiliations:** 1 Avenir Health, Glastonbury, CT USA; 2 University of Nairobi, Nairobi, Kenya; 3 LVCT Health, Nairobi, Kenya; 4 FHI 360, Washington, DC USA; 5 Columbia University, New York, NY USA; 6 National AIDS and STI Control Programme, Nairobi, Kenya; 7 County Government of Kakamega, Kakamega, Kenya; 8 Jhpiego, Nairobi, Kenya

**Keywords:** Cost, HIV prevention, PrEP, PrEP ring, Willingness to pay, Contingent valuation

## Abstract

**Background:**

As Kenya prepared to introduce the PrEP ring (a long-acting product used by women for HIV prevention), the need to understand the resources required became increasingly important. The aim of this study was to determine the costs and preferences of potential ring clients by conducting a normative cost analysis and a contingent valuation study (In the context of willingness to pay literature, “preference” is used to refer to an interest in a service or product with specified benefits).

**Methods:**

The study incorporates two parts: 1) a normative costing to estimate potential costs of providing the PrEP ring and 2) a willingness to use/pay assessment to evaluate client preference for the PrEP ring. Oral PrEP program managers from 12 facilities were interviewed to assess the direct and indirect resources required to deliver PrEP ring services. 539 women were interviewed (including both younger and older women, as well as female sex workers) using a questionnaire to assess the strength of the expressed interest in PrEP ring use, as reflected by their contingent valuation of the product. Women were presented with payment cards from which they selected the most they would be willing to pay. The primary outcomes of the study were: 1) the annual cost of PrEP ring use and 2) the average willingness to pay for the PrEP ring. Women in the willingness to pay component were selected, using a convenience sample, with approximately equal numbers of oral PrEP users and clients from other health services.

**Results:**

The cost to provide a full year of PrEP ring was US$206; 76% (US$156) of that cost was attributed to the PrEP rings. Of the respondents, 78% indicated some interest in using the PrEP ring; among those interested, 83% indicated some willingness to pay for it. Single women and women currently using oral PrEP expressed more interest in using the ring than women who were married or were not currently oral PrEP users. The median willingness to pay per visit was US$1.86.

**Conclusions:**

This analysis revealed that costs are predominantly driven by commodities. Attempts to further reduce the cost of the commodities could significantly reduce the overall cost of PrEP ring service.

Approximately half the women were willing to pay up to US$2 per month for the PrEP ring. Since the demand for the PrEP ring appears to be higher among current oral PrEP users compared to non-users, women who have initiated oral PrEP but who are unable or unwilling to continue may be good candidates for PrEP ring use. The annual willingness to pay for the PrEP ring (US$11.16) was significantly lower than the ring’s cost ($206 per year), which suggests that attempts to fully recover costs would present a significant barrier for Kenyan women and would minimize societal benefits.

## Introduction

UNAIDS estimates that in 2021, 38.4 million people were living with HIV and, in part due to the inability to achieve prevention targets, 1.5 million people acquired HIV, well above the 500,000 target for new HIV infections set in 2016 [1]. Women and girls accounted for about 53% of all people living with HIV globally and 63% of all new HIV acquisitions in sub-Saharan Africa [2]. Pre-exposure prophylaxis (PrEP) promises to reduce HIV incidence, but the first method approved and available since 2012, daily oral PrEP, remains underutilized [3]. By the end of 2021, 2 million people had initiated PrEP, which significantly missed the fast-track target of 3 million people on PrEP by 2020 [3]. Women specifically have relatively lower initiation and higher discontinuation rates on PrEP [4].

Kenya’s HIV prevalence among adults 15–49 years peaked at an estimated 10–11% in the mid-1990s and declined to about 4.9% in 2018 [5]. Annual new HIV acquisitions decreased from an estimated 71,000 in 2010 to about 33,000 in 2020 [4]. This substantial reduction in HIV incidence has been achieved through a combination of approaches, including the scale-up of HIV treatment and viral suppression, condom promotion and distribution, voluntary medical male circumcision (VMMC), and oral PrEP. Even with the availability of these preventative interventions, many remain at risk of HIV exposure, and incidence has not declined uniformly across populations and geographic areas in Kenya. In 2017, 52,767 Kenyans acquired HIV, with young people ages 15–24 accounting for 33% of all new HIV acquisitions [6]. Women continue to be disproportionately affected, with new HIV acquisitions among young women ages 15–24 in 2018 more than double those among young men of the same ages (11,000 vs 5,000) [7].

Kenya has been a leader in sub-Saharan Africa in the approval and introduction of novel biomedical HIV prevention interventions, being one of the first countries to incorporate VMMC, daily oral PrEP, and most recently, regulatory approval for the PrEP ring. VMMC was implemented as part of the country’s HIV prevention strategy in 2008; by 2016, 92.6% of men in targeted counties were circumcised [8]. Daily oral PrEP received regulatory approval in 2016 and was rolled out nationally in 2017, with a commitment to make it available to 500,000 people at high risk of HIV infection by 2020 [9]. However, by 2020, only 52,229 individuals had received at least one dose of oral PrEP.

The PrEP ring is a female-initiated product that reduces the risk of acquiring HIV by releasing the antiretroviral (ARV) drug dapivirine locally in the vagina. An inserted ring releases dapivirine for one month, providing users with discreet, long-acting protection from HIV infection [10]. A systematic review and meta-analysis of PrEP ring trials demonstrated that the ring is effective in reducing the risk of acquiring HIV infection. Two randomized controlled trials — the Ring Study (IPM-027) [11] and ASPIRE (MTN-020) [12] — reported that the dapivirine ring was approximately 30% effective in reducing HIV infection in intention-to-treat analysis. The results from two-open-label extension studies, DREAM and HOPE, found increased efficacy, increased adherence, and increased retention relative to the randomized control trials [13, 14]. The results from one of the open-label extension studies indicated a 62% reduction in HIV transmission, comparing study results to a simulated control [14]. The World Health Organization has recommended the ring for adoption as part of a combination prevention package since 2021 [15], citing evidence from South Africa that the PrEP Ring may be a cost-effective intervention [16–18].

As the reduction in the number of new HIV infections in Kenya continues to slow, efforts to achieve greater gains may be constrained by the limited funding available for prevention activities and may require new approaches to service provision. The hope is that including the PrEP ring as an additional, alternative prevention option (and not a replacement) for Kenyan women will increase overall coverage of HIV prevention, leading to greater reductions in HIV incidence.

This study has two primary aims. It first seeks to assess the normative unit cost of delivering the PrEP ring in various service delivery models (e.g., nongovernmental organizations [NGOs], private and public health facilities) and for various populations (young women ages 18—24, women older than 24, and female sex workers). A normative costing approach was chosen because the PrEP ring was not approved or available in Kenya at the time of data collection. The second purpose of the study was to evaluate how much potential PrEP ring clients might be willing to pay for the product. Insights into the costs of delivering the PrEP ring and the potential willingness to pay could inform decision-making on the promotion and distribution of the ring as part of Kenya’s combination prevention efforts.

## Methods

### Site recruitment and setting

The research team utilized a convenience sample of 12 facilities located in four Kenyan counties: Nairobi, Migori, Kisumu, and Kisii (Table 1). Six of the facilities were in Nairobi, three in Migori, two in Kisumu, and one in Kisii. All selected sites had been offering oral PrEP for at least six months and were therefore familiar with PrEP and the methods of delivering these services. (As noted earlier, none of the sites were offering the PrEP ring because it had not yet been approved for use in Kenya).

**Table 1:** Selected study sites

County	Site Name	Target Population	Project	Type of Site
Nairobi	BHESP drop-in center (DICE)	FSWs	DARAJA	NGO
Nairobi	Sokoni DICE	FSWs	DARAJA	NGO
Nairobi	Kware DICE	FSWs FP clients (25–44 years)	DARAJA	Integrated MOH/NGO
Nairobi	Lunga Lunga Health Center	AGYW family planning (FP) clients (25–44 years)	N/A	Integrated MOH/NGO
Nairobi	Mukuru Health Center	AGYW FP clients (25–44 years)	N/A	Integrated MOH/ NGO
Nairobi	Pumwani Health Center	FSWs Women (25–44 years)	N/A	MOH
Kisii (Lake Region)	Tekeleza	FSWs FP clients (25–44 years)	STEPS	Integrated MOH/NGO
Kisumu (Lake Region)	Nyamgóma	FSWs FP clients (25–44 years)	STEPS	Integrated MOH/NGO
Kisumu (Lake Region)	Nightingale Medical Center	Women (25–44 years)	N/A	Private, faith-based
Migori (Lake Region)	Isebania DICE	FSWs	STEPS	NGO
Migori (Lake Region)	Kakrao DREAMS site	AGYW	STEPS	NGO
Migori (Lake Region)	Ragana–Oruba DREAMS site	AGYW	STEPS	NGO

The sample size was determined based on the need to obtain variation in the types of facilities where data was collected, while being limited by the budget available. Cost data were collected from sites selected for the study and included information on a mix of facility types in the two regions of Kenya (Nairobi and the Lake Region). In total, 12 facilities were costed. All 12 facilities were selected because they were currently providing oral PrEP and were in one of five counties (Nairobi, Kisii, Kisumu, Migori and Homa Bay).

In Nairobi, the study utilized three sites focused on PrEP service delivery for female sex workers (FSWs) within the DARAJA project (Developing Risk Awareness towards Joint Action) [19]. DARAJA is an HIV prevention project implemented by LVCT Health that aims to increase access to and availability of sustainable, high-quality, comprehensive health services and interventions for key populations and others in need of HIV prevention. The project provides comprehensive HIV prevention services (including oral PrEP) to FSWs through drop-in centers. The three other sites in Nairobi were selected to provide additional perspectives from health facilities (two Ministry of Health [MOH] and one faith-based site).

In the Lake Region (Kisumu, Kisii, and Migori counties), the study selected a total of six sites. Five of the sites (two sites focused on adolescent girls and young women [AGYW] and three focused on FSWs) were selected from within the Support Towards Expanded Prevention Services (STEPS) project [20]. The STEPS project provides comprehensive HIV prevention services (including oral PrEP) to FSWs at drop-in centers. STEPS also includes sites where LVCT Health is providing services through the Determined, Resilient, Empowered, AIDS-free, Mentored, and Safe (DREAMS) project to reach women and girls ages 10–24 years. The program provides safe spaces and links to health facilities where evidence-based HIV interventions, including oral PrEP, are offered. The sixth site in the Lake Region is a private-sector facility.

### Ethical approval and consent to participate

The Ethics and Scientific Review Committee of AMREF in Nairobi Kenya (AMREF-ESRC P915/2020) approved the study on March 29, 2021, and the Office of International Research Ethics at FHI 360 in Durham, North Carolina (study number 1686619), provided a human subjects exemption for this research on June 17, 2021. A health economic analysis plan was developed and is available upon request.

### Informed consent

All study participants were provided with information on the scope and nature of the interviews to be conducted. Written consent was obtained by the data collectors from all participants in the study. During the informed consent process, data collectors explained to eligible client and stakeholder participants the basic purpose and conduct of the study, including confidentiality procedures and the right to refuse or withdraw at any time. Data collectors read the informed consent form to every participant and gave the participant time to ask questions about the study. Data collectors asked participants if they were willing to participate in the study and asked the participants to sign their name on the informed consent. All participants were offered a paper copy of the form which included the contact information for FHI360 and AMREF’s IRB. If the participant did not agree to sign the form, the interviewer discontinued the process and moved on to the next eligible individual.

### Costing

#### Costing approach

The study adopted an economic costing approach that focused on total site level costs to the private and public health care systems for delivering the PrEP ring. Costs were calculated for personnel (both direct and indirect) at the facility, the rings, other consumables, equipment/furniture, and overhead (e.g., electricity, water, etc.). Above-site-level costs were not collected. Interviews were conducted with program managers responsible for managing the oral PrEP program at each of the 12 health facilities to assess how they would deliver the PrEP ring based on service delivery norms. Norms were identified based on the type of visit made by the client. The “initiation visit” would involve reception into the facility and triage, including a rapid HIV test, meeting with the identified clinical staff member for participation in a relevant health education session and counselling, and being provided with one ring. “Refill visits” would occur at the end of months 1 and 2, with clients receiving a short clinical session to identify any challenges and receive one ring (HIV tests were assumed not to be required during these refill visits.). Finally, “quarterly visits” would be conducted at the end of months 3, 6, and 9. During each of these quarterly visits, HIV tests would be performed, and clients would receive a three-month supply of rings. As clients were assumed to insert the ring at home, no additional procedures were assumed to be required in the delivery of PrEP ring services (other than the rapid HIV testing performed during initiation visits and quarterly visits). Estimated capital costs and other overhead costs were distributed to PrEP ring clients based on a comparable distribution of costs for providing oral PrEP to clients. To allocate fixed costs, we assumed that the number of PrEP ring clients would be the same as the number of oral PrEP clients. No assumptions were made about whether PrEP ring clients would represent “new” clients or whether they would come from existing oral PrEP clients.

#### Data collection

Costs were collected from the 12 selected health facilities that were already delivering oral PrEP services. The normative costing approach followed assumptions regarding the various potential models for delivering PrEP ring services from the perspective of the health system in Kenya. Data was collected from all 12 sites between April 2021 and June 2021.

The main direct costs collected included consumables (HIV test kits, gloves, etc.), the PrEP rings, and the salaries of any personnel directly involved in the delivery of services, e.g., clinical officer, nurses, and counselors. The cost of the ring has not yet been confirmed in Kenya. However, information from the manufacturer at the time of this analysis indicated that the likely cost would be US$13 per ring, which includes not only the rings themselves but also all associated supply chain costs required to deliver the product to health facilities.

The costs were calculated for initiation visits, refill visits, and quarterly visits. Program managers were asked to estimate the time required for each clinical staff member to deliver the PrEP ring, including the time for counselling. This allowed for a capacity cost rate to be calculated for each clinical staff member to identify the cost per minute of clinical time. Since the service was not being provided at the time of this analysis, it was not possible to conduct a time-and-motion study to calculate the time required for delivering the service. The staff salaries (including benefits) of NGO and private sector personnel were collected from each facility. Estimated government salaries were based on existing 2020 government pay scales and were obtained from salaries and benefits tables provided by government sources.

Indirect costing data collected include capital items (equipment, furniture, etc.), maintenance and utility costs (water, electricity, etc.), support personnel (staff providing support services), and management/supervision. All capital costs were discounted at a rate of 3%. To allocate indirect costs to PrEP and non-PrEP services, staff at each facility determined the proportion of their space and time that was being used for the delivery of PrEP services. For example, if a specific space was used 50% of the time for PrEP services and comprised 40% of a facility’s space, then 20% of all indirect costs were assigned to PrEP.

All cost data were collected in Kenyan shillings and were converted into 2021 US dollars using an exchange rate (as of May 2021) of KSh107.5 = US$1 [21]. Cost data were entered into a separate spreadsheet for each facility. The data were evaluated by both a health economist at the University of Nairobi and a health economist at Avenir Health to identify any inconsistencies.

#### Data analysis

Once cost data collection had been completed in each of the 12 facilities, the costing team went through a process of data cleaning and validation, which included identifying any missing data and assessing inconsistencies in responses. Where further data cleaning was necessary, the lead consultant contacted program managers to address any gaps or inconsistencies. As this study was based on a normative costing exercise, it was necessary to predict how services would be delivered for the PrEP ring and to assign costs to each step in the delivery of these services. The outcome variable analyzed for the costing component was the estimated cost per visit and cost per year (assuming 12 months of PrEP use) for PrEP ring clients, broken down by initial visits, refill visits and quarterly visits. In addition, the unit cost per year is further subdivided into NGO facilities, private hospitals, and public health centers.

### Willingness to pay

#### The WTP approach

The willingness to pay (WTP) study was performed using a questionnaire (see Supplementary Material) that was developed using a multistage, iterative process involving health economists at the University of Nairobi and Avenir Health. The questionnaire was adapted from a costing study of oral PrEP completed under the Jilinde Project, funded by the Bill and Melinda Gates Foundation, which supported oral PrEP rollout in Kenya [22]. The WTP questionnaires and client informed consent form were translated into Kiswahili, Kisii, and Luo, and then back translated to verify the quality of the translation. The WTP questionnaire was pilot tested at a pre-determined site in Nairobi that was not a part of this study. After the piloting, the questionnaire was reviewed and reworked based on the feedback received from participants and the experience of the research associates.

In terms of the women interviewed, they were selected based on their willingness to participate in the study and sign the informed consent form. The women were receiving health services at one of the 12 facilities, although they could either be current oral PrEP clients or clients for other health services. To be eligible, women needed to fit within one of three risk groups (AGYW between 18 and 24 years old, women at-risk between 25 and 44 years old or female sex workers).

The facility in-charge (or managers, as appropriate), as well as other site personnel, were informed about the study and worked with the research associates to identify potential clients for interviews. Clients were selected based on being female and being outpatients at the respective facility. All respondents were either current users of oral PrEP or were not using oral PrEP but perceived themselves to need an HIV prevention method. Women were not asked if they preferred oral PrEP or PrEP ring. Women were selected by research associates using a convenience sample of women who were currently engaged in the oral PrEP program or enrolled in other health services. Staff intentionally conducted a convenience sample of women, recruiting comparable numbers of female oral PrEP clients and female clients who were attending for other health services. If the number of clients from one subpopulation (e.g., oral PrEP clients) was achieved, data collection then focused on only the second subpopulation (e.g., clients of other health services).

The research associates briefly explained the study to each client, using an approved recruitment script, and the client was asked if she was interested in participating in an interview. Those who said yes or were interested in learning more were given an informed consent form. Interviews were conducted in a space with sufficient privacy so that they would not be overheard. Each interview took approximately 30–45 min, and research associates remained at each facility for approximately one week.

#### Data collection

The questionnaire was implemented in four different sections. The first section involved collecting simple data related to the facility and the services being accessed by each respondent. In the second section, research associates detailed much of the socio-economic and demographic data related to each respondent, including education, employment, relationship status, and household information. In section three, they probed respondents’ knowledge of HIV prevention, especially oral PrEP. This section also introduced the PrEP ring to respondents. As this product was not yet available in Kenya, we utilized the opportunity to help respondents understand the ring, how it works, how it is used, and how effective it may be at preventing HIV infection if used properly. The fourth section of the questionnaire dealt directly with estimating willingness to use and willingness to pay for the PrEP ring.

Respondents were first asked if they would potentially be interested in using the PrEP ring if it became available in Kenya. For those potentially interested in the PrEP ring, they were then asked if they had to pay for the ring, what would be the maximum amount they would be willing to pay monthly. Respondents who indicated a potential interest in the PrEP ring were also asked if they would prefer the PrEP ring, oral PrEP, or both. If a respondent indicated that they would not be willing to pay for the ring, then they were asked for the reason. If the response was that the participant would not be able to afford purchasing the ring at any price, then it was determined that these responses represented a “true zero.” However, if a respondent indicated that they would not be willing to pay for the ring because other interventions (including oral PrEP) were being offered for free and that the ring should be available free of charge as well, then this response represented a “protest zero.” In other words, the respondent was protesting having to pay for the service. These responses were excluded from the final estimate of median willingness to pay [23, 24].

If the respondent did indicate a willingness to use the PrEP ring, the study questionnaire then employed a “payment card format” to estimate the amount each respondent was willing to pay. This method has been used widely, enjoys high response rates, is good for studies with smaller sample sizes, produces results that are well aligned with an individual’s ability to pay, and generally avoids anchoring bias better than other approaches [25, 26].

Using the payment card format, research associates presented each respondent with a set of 10 cards with different values on them ranging from KSh50 to KSh4,000 (approximately US$0.47 to US$37.21,). The respondent was asked to indicate the card with the highest amount they would be willing to pay for the PrEP ring every month. Once the respondent had selected a card, they were then asked by the interviewer if they would be willing to pay the next highest amount. If the respondent said “yes,” then the card with the next highest amount was selected, and the process was repeated. If the respondent said “no,” the last card selected became the highest amount the respondent would be willing to pay for the ring.

To ensure that respondents understood the value of what they were being asked to pay for and what influence this knowledge had on the amounts they indicated being willing to pay, the questionnaire varied the effectiveness of the PrEP ring [21, 27]. Half of the questionnaires indicated that the ring reduced the chances of HIV infection by one-third; the other half of the questionnaires indicated that the ring reduced the chances of infection by one-half. Questionnaires indicating the different levels of effectiveness were alternated so that the study would have an equal number of participants responding to the two different levels of effectiveness.

#### Data analysis

The data analysis focused on obtaining the median willingness to pay per visit for women responding to the willingness to pay questionnaire. In addition, there was an extensive statistical analysis to determine the associations between willingness to pay (and willingness to use) the PrEP ring and other variables using logistic regression.

Dependent variables were coded as binary responses, with individuals willing to pay non-zero amounts classified as willing to pay. Age and monthly income were transformed into categorical variables. The categories considered for age were 15-to-19 years, 20–24 years, 25–34 years, and 35 + years and quartiles were used to create categories of the monthly income. Incomes less than the first quartile were classified as “low,” those between the first and the third quartiles were coded as “medium” and those above the third quartile were coded as “high.” Multivariate logistic regressions were used to assess the effects of co-factors on the willingness to pay and willingness to use the PrEP ring. The analyses were performed with R version 3.5.1 (R Core Team, 2021).

## Results

### Cost

#### Cost per visit

**Fig. 1:**
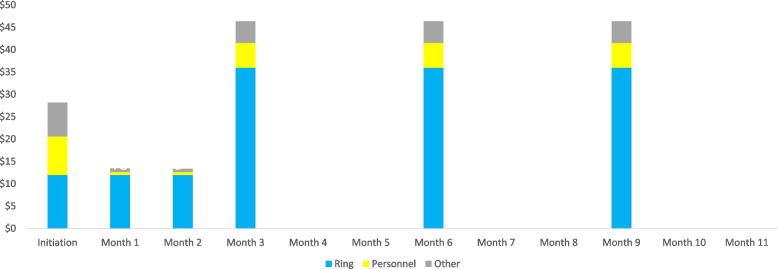
Unit cost by visit

The unit cost for initiation visits was determined to be US$29 (Fig. 1). The factors most influencing the cost included the PrEP ring, consumables required to initiate new clients, personnel time required for the delivery of services, and HIV tests performed. The direct personnel costs for these initiation visits were the highest of any visits because they require substantial amounts of time for providers to explain the use of the PrEP ring and conduct an HIV test. The refill visits in months 1 and 2, which require no HIV tests and only limited clinical time, were relatively less expensive, with a unit cost of US$14 per visit. The quarterly visit unit cost was US$49 each (including US$39 for three rings). The higher cost for these visits was due to clients receiving three rings and an additional HIV test.

The cost of a year of PrEP ring, if clients continue using the product for an entire year, was estimated to be US$206 (excluding demand creation costs and other above-site costs). Of this amount, 76% was for the ring. (This estimate assumed US$13 per ring, or US$156 per year.) The average total time (triage, testing and consultation) was 92 min for initiation visits, 10 min for refill visits and 59 min for quarterly visits.

Clinical personnel were the next highest costs, estimated to be US$26.48 per year, or 14% of all costs. Other drivers of cost in descending order were support personnel (US$7.74 per year, or 4% of all costs) and other remaining costs (US$16 per year, or 8% of all costs), which were comprised of other consumables (including HIV test kits), non-consumables, capital equipment, building maintenance, and management/supervision.

#### Cost by type of facility

**Fig. 2:**
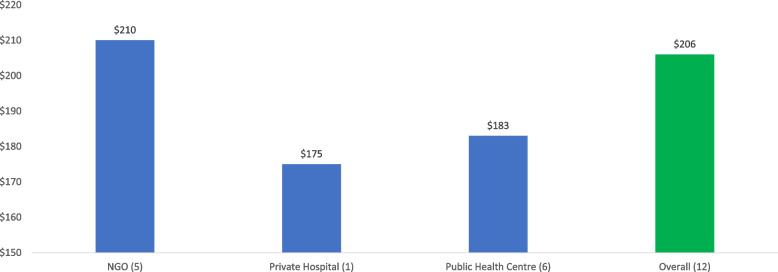
Cost per client per year by type of facility^a^. ^*a*^The figures in parentheses represent the number of sites that were costed. All assume a perfect continuation rate over a 12-month period

Figure 2 provides the cost based on the type of facility. The most expensive facilities are those operated by NGOs, at a cost of US$210 per client per year, followed by the public health facilities, with an average cost of US$183 per client per year. The least expensive is the private for-profit sector, although it should be noted that this cost estimate represents only one facility.

#### Cost in year 2

Continued use of the PrEP ring beyond year 1 would have several cost advantages, including shifting from six visits per year to only four quarterly visits. Based on these assumed number of visits, the annual cost of the PrEP ring would decline from US$206 to US$198 per year.

#### Sensitivity analysis

The study also assessed the potential costs under varying assumptions regarding the number of visits required and the cost of the rings. The costed program assumed one initiation visit, two refill visits, and three quarterly visits per year. If one of the refill visits were eliminated and clients began having quarterly visits after month 1, the total cost would decline by just one dollar per year, from US$206 to US$205 per year; although the non-ring costs in month 1 would be skipped, the ring costs would not change. This relatively small decline in costs might occur with a significant benefit to women, reducing the number of times they would need to visit a clinic from six to five times per year.

In addition, the cost of the ring was based on an estimate made at the time of the study. If the cost is assumed to be US$15 per ring, then the total cost per year would rise from US$206 per year to US$230 per year.

### Willingness to use/pay

A total of 539 women participated in the WTP survey, ranging in age from 18 to 58 years, with a median age of 24. More than half of respondents (53%) were ages 18–24, 22% were 25–29, and 14% were 30–34. Those in the older age groups of 35–39 and 40 + years, represented 6% and 4% of the total number (Table 2).

**Table 2:** Demographic variables for WTP respondents

		%	n
AGE	18–24	53%	286
	25–29	22%	121
	30–34	14%	78
	35–39	6%	34
	40 +	4%	20
URBANIZATION	Urban	89%	478
	Peri-urban	3%	18
	Rural	8%	43
COUNTY	Nairobi	46%	247
	Migori	28%	152
	Kisumu	18%	98
	Kisii	8%	42
MARITAL STATUS	Never married/single	254	47%
	Married/in union	122	23%
	Widowed/divorced	163	30%
LEVEL OF EDUCATION	None/primary	171	32%
	Secondary	290	54%
	College/tertiary	78	14%
CURRENTLY USING ORAL PrEP	No	260	48%
	Yes	279	52%

The great majority of respondents were from urban areas (89%), with 247 (46%) from Nairobi, 152 (28%) from Migori, 98 (18%) from Kisumu, and 42 (8%) from Kisii. Most respondents were single and had not married (47%), and the majority had at least some level of secondary education (54%), while only 14% had some college or tertiary-level education. Fifty-two percent of the respondents were currently using oral PrEP while 48% were not.

Of the total number of participants, 78% (*N* = 423) responded “yes” or “maybe” they would be interested in using the PrEP ring, while 116 participants, or 22%, had no interest in using the ring. Out of those who indicated they were interested in using the ring, 83% were willing to pay something for it, and 17% said they would not pay for it. The amount that women were willing to pay per month ranged from as low as US$0.47 to as high as US$27.91. The median amount all study participants were willing to pay per visit was US$1.86. Among women potentially interested in the PrEP ring, 77% indicated that they preferred the PrEP ring, 8% indicated that they still would prefer oral PrEP and 15% indicated that they would prefer to use both.

Table 3 displays the effects of all the dependent variables on the willingness to use the PrEP ring included in the analysis. It shows that, in a bivariate analysis, married/in-union women were less likely to use the PrEP ring than never married/single women (*P* = 0.05). On the other hand, women who were employed full-time were much more likely to indicate they would adopt the PrEP ring compared to unemployed women (*P* = 0.00). Women with medium income were much more likely to indicate they would adopt the PrEP ring compared to women with a low income (*P* = 0.05). In addition, women who were currently using oral PrEP were more likely to be willing to adopt the PrEP ring as opposed to women who were not currently on oral PrEP (OR = 1.8, *P* = 0.00).

**Table 3:** Effects of cofactors on willingness to use the PrEP ring

		Bivariate OR (95%CI, P-Value	Multivariate OR (95%CI), P-Value	Number (%)
Age	< 25	1	1	286 (53%)
	25–29	1.3 (0.8–2.1), *P* = 0.37	1.5 (0.8–2.7), *P* = 0.21	121 (22%)
	30–34	1.3 (0.7–2.5), P = 0.37	1.5 (0.7–3.1), P = 0.34	78 (14%)
	35 +	1.6 (0.7–3.3), P = 0.26	2.2 (0.9–5.4), P = 0.09	54 (10%)
Marital Status	Never married/single	1	1	254 (47%)
	Married/in-union	0.6 (0.4–1.0), P = 0.05	0.5 (0.3–0.9), P = 0.01	122 (23%)
	Widowed/divorced	0.9 (0.5–1.4), P = 0.52	0.4 (0.2–0.8), P = 0.01	163 (30%)
Level of Education	None/primary	1	1	171 (32%)
	Secondary	0.9 (0.6–1.5), P = 0.71	1.1 (0.6–1.9), P = 0.71	290 (54%)
	College/tertiary	0.7 (0.4–1.4), P = 0.30	1.0 (0.5–2.0), P = 0.93	78 (14%)
Employment Status	Unemployed	1	1	177 (33%)
	Employed full time	2.1 (1.3–3.3), P = 0.00	2.0 (0.8–5.1), P = 0.17	93 (17%)
	Employed part time	1.1 (0.6–2.0), P = 0.67	1.0 (0.4–2.7), P = 1.00	269 (50%)
Total Monthly Earnings	Low	1	1	150 (28%)
	Medium	1.6 (1.0–2.6), P = 0.05	1.3 (0.5–3.2), P = 0.64	252 (47%)
	High	1.6 (0.9–2.9), P = 0.08	1.1 (0.4–3.2), P = 0.91	131 (25%)
Amount spent on transport (Ksh/100)	1.0 (0.6–1.8), P = 0.98	0.9 (0.5–1.6), P = 0.67	Amount spent on transport (Ksh/100)	
Currently using oral PrEP	No	1	1	280 (48%)
	Yes	1.8 (1.2–2.8), P = 0.00	1.7 (1.0–2.6), P = 0.03	259 (52%)
Efficacy level	One-half	1	1	270 (50%)
	One-third	0.8 (0.5–1.2), P = 0.28	0.7 (0.5–1.1), P = 0.14	269 (50%)

Results from the multivariate analysis suggest that women already using oral PrEP were much more likely to use the PrEP ring (*P* = 0.03). In addition, women who were married or in-union, or those widowed or divorced, were less likely to want to use the PrEP ring compared to never married women (*P* = 0.01). In both the bivariate and the multivariate analyses, the indicated efficacy levels of the PrEP ring (either reducing infection by half or one-third) did not influence the willingness of the respondent to use the product. In the multivariate analysis, women’s full-time employment status was no longer a significant factor in determining whether they had interest in adopting the PrEP ring (P = 0.17).

Table 4 displays the effects of all the dependent variables included in the analysis on the willingness to pay for the PrEP ring. It shows that, in a bivariate analysis, the willingness to pay for the PrEP ring was about 70% and 80% higher for women ages 25–29 (*P* = 0.02) and 30–34 (*P* = 0.04), respectively, compared to those younger than 25 years.

**Table 4:** Effects of cofactors on willingness to pay for the PrEP ring

		**Bivariate OR (95%CI), P-Value**	**Multivariate OR (95%CI), P-Value**
**Age**	< 25	1	1
	25–29	1.7 (1.1–2.7), *P* = 0.02	1.3 (0.8–2.3), *P* = 0.34
	30–34	1.8 (1.0–3.1), *P* = 0.04	1.1 (0.5–2.1), *P* = 0.08
	35 +	1.6 (0.9–3.0), *P* = 0.14	1.3 (0.6–2.8), *P* = 0.48
**Marital status**	Never married/single	1	1
	Married/in union	0.9 (0.6–1.4), *P* = 0.72	0.7 (0.4–1.3), *P* = 0.27
	Widowed/divorced	1.5 (1.0–2.3), *P* = 0.06	0.7 (0.4–1.3), *P* = 0.29
**Level of education**	None/primary	1	1
	Secondary	0.8 (0.5–1.1), *P* = 0.20	1.0 (0.6–1.7), *P* = 0.89
	College/tertiary	0.9 (0.5–1.6), *P* = 0.78	1.5 (0.8–2.8), *P* = 0.23
**Employment status**	Unemployed	1	1
	Employed full time	3.4 (2.3–5.2), *P* = 0.00	1.7 (0.7–4.2), *P* = 0.22
	Employed part time	1.3 (0.8–2.2), *P* = 0.26	0.7 (0.3–1.7), *P* = 0.38
**Total monthly earnings**	Low	1	1
	Medium	2.5 (1.7–3.8), *P* = 0.00	2.1 (0.9–5.1), *P* = 0.09
	High	3.3 (2.0–5.5), *P* = 0.00	2.4 (0.9–6.4), *P* = 0.09
**Amount spent on transport (Ksh/100)**		1.1 (0.7–1.8), *P* = 0.63	0.9 (0.5–1.5), *P* = 0.63
**Currently using oral PrEP**	No	1	1
	Yes	1.8 (1.2–2.6), *P* = 0.00	1.5 (1.0–2.2), *P* = 0.04
**Efficacy level**	One-half	1	1
	One-third	0.9 (0.7–1.3), *P* = 0.76	0.9 (0.6–1.3), *P* = 0.52

Among women who were willing to pay something for the PrEP ring, women employed full time were willing to pay for the ring more than three-fold than those who were unemployed (*P* = 0.00). Those with medium (*P* = 0.00) or high income (*P* = 0.00) were willing to pay 2.5 to 3.5 times more than low-income women (an expected result, since willingness to pay should be closely associated with ability to pay). Women currently using oral PrEP were willing to pay 80% more than women who were not currently using oral PrEP (*P* = 0.00).

Results from the multivariate analysis suggest that women who were already using oral PrEP were willing to pay 50% more than women who were not current using oral PrEP (*P* = 0.04). This variable was the only independent variable that was statistically significant.

## Discussion

The PrEP ring offers a unique, convenient, discreet, and user-controlled approach to HIV prevention. This technology is a useful prevention tool in countries where women are highly vulnerable to HIV exposure. Yet in many countries, the PrEP ring is currently not available despite increased access to oral PrEP. One of the reasons for this limited availability is the lack of knowledge regarding the cost required for countries to commit to providing access to PrEP rings.

The analysis revealed that the annual cost of providing the PrEP ring will be US$206 per woman per year. As noted, about three-quarters of this cost is related to the PrEP ring itself. There was some variation in costs by type of facility but given the relatively small sample size (12 sites), it was difficult to draw absolute conclusions about relative costs. The greatest variations were observed in personnel costs, which did vary widely from facility to facility.

The willingness to use and willingness to pay analyses provided some interesting findings about individual preferences and the strengths of those preferences. The responses to both the willingness to use and the willingness to pay questions revealed a stronger preference for the PrEP ring among current oral PrEP users compared to non-users. This may suggest that women already using oral PrEP believe they need HIV prevention and are therefore interested in products that provide protection. The finding may also suggest that the demand for the PrEP ring may come, at least initially, from existing oral PrEP users. It could potentially be detrimental for fully adherent women to shift from oral PrEP to PrEP ring. Adopting the PrEP ring would be most beneficial for women who are not currently PrEP users and for women who wish to combine oral PrEP and PrEP ring.

The analysis also revealed that married women had less interest in the PrEP ring than single women. However, married women who were interested in the PrEP ring had a much stronger preference for the PrEP ring (as represented by their willingness to pay) compared to single women. This finding may suggest that single women will be the early adopters, but married women may eventually be as likely to sustain PrEP ring use as their single counterparts.

It remains unclear if there is a stronger preference for the PrEP ring among those who are higher earners or those who are lower earners. The results of the bivariate analysis suggest middle- and high-income women were likely to adopt the PrEP ring compared to low-income women. If this is the case, demand creation efforts might be needed to promote the PrEP ring among the poorest women. The bivariate analysis for willingness to pay also showed a stronger preference for the PrEP ring among medium- to higher-income women, but this is likely driven by wealthier women’s greater ability to pay and not just preference.

The potential use and strength of that preference for the PrEP ring are not likely to vary by level of education. Thus, demand for PrEP ring may be similar among women with high levels of education and women with low levels of education.

Finally, the analysis revealed that the preference for the PrEP ring, as well as the strength of that preference, were not affected by the assumed efficacy of the product. This is somewhat surprising, given that one would assume women would prefer a more efficacious product over a less efficacious one. It was hypothesized that the women who were told that the PrEP ring reduces infections by one-half would have more interest in the product than the women who were told that the product would reduce infections by only one-third. This finding may suggest that women had difficulty understanding the relevance of the efficacy data that was provided. Alternatively, it may suggest that the two efficacy figures were so close that they were essentially indistinguishable in the minds of the respondents.

### Study limitations

The analysis for this normative costing study is based on the expected costs of providing services, and not the actual observed use of resources required to provide the PrEP ring. Various factors may make the actual delivery of services either more or less expensive than the normative costing would suggest. For example, the normative costing is based on the estimated amount of clinical staff time required to provide the PrEP ring. In addition, adding the PrEP ring to sites that are already offering oral PrEP might affect the unit cost of both services (for example, introducing new PrEP users might allow facilities to reduce existing downtime while allocating fixed costs over a larger number of clients).

The estimated cost of the PrEP ring (including supply chain costs) was determined to be $13 per ring. This cost, as indicated, represents an essential cost driver. The actual cost of delivering the rings was based on the best estimate of the manufacturer and may differ in any final negotiations between the country and the manufacturer. This could potentially have important unit cost implications.

The costing study was not able to address continuation rates or the cost of wastage. This analysis did not assess if PrEP rings might have expired at a facility or alternatively might have been accepted by women but not used. Also, because this study focused on the estimated minutes required for providing PrEP ring, it did not consider how “downtime” by the service provider was incorporated into the cost analysis. Thus, for example, if a staff member spends an hour with a client, but due to a lack of demand that client is the only client seen by the provider in a day, then the analysis would cost the one hour spent seeing patients and not the seven hours of downtime. In addition, costs may differ depending on the approach to providing the PrEP ring (e.g., multi-month dispensing of rings might start earlier than the end of month 2). Finally, the actual cost of the commodities may vary based on negotiations with the manufacturer.

Participants in the WTP study were recruited only from women attending clinics (either an oral PrEP clinic or other health clinics), and the sample size was relatively small, so we did not have enough power to detect all the potential associations and interactions. Therefore, we confined ourselves to variables deemed most important in our context and reduced the complexity of our analyses to a simpler model. However, the results provide an indication of women’s willingness to use and pay for the ring. In addition, the study was not designed to address potential social desirability bias. Given that willingness to pay was based on verbal reports and not actual payment, the results may be subject to overestimation of women’s actual willingness to pay.

In addition, the researchers did not collect any data on the number of women who refused to participate in the willingness to pay analysis. Also, no data was collected on the number of women who were initially selected but determined to be ineligible (e.g., women who were under 18 years old or over 44 years old).

Additional, in-depth analyses are needed to increase our understanding. First, it would be useful to cost the rollout of the actual PrEP ring implementation (including a time-motion component), as opposed to conducting a normative costing of the ring. At the time of submission, the CATALYST study is analyzing the actual cost of rollout when women are provided a choice between oral PrEP, the PrEP ring, and CAB PrEP. How women respond to having a choice of PrEP methods will provide even more insights into the resources required and the benefits of allocating those resources across a range of PrEP choices.

## Conclusions

Understanding both the cost of PrEP and the preference for oral PrEP and the PrEP ring provides an opportunity to better implement a program that can offer choice to women who need HIV prevention. As demonstrated here, a range of factors need to be considered in implementing the PrEP ring, including understanding the budgetary requirements and the likely preferences of women who have the option to choose from both forms of PrEP.

Offering the PrEP ring will involve an incremental investment of resources above those required for oral PrEP alone. The exact cost of scale-up will depend on several assumptions, including the extent to which the PrEP ring will expand the total number of PrEP users, the locations where the ring is offered, and the uptake of both services. While the real-world costs remain somewhat uncertain, this cost analysis does provide a useful indication of the potential costs of providing PrEP rings for a year as the Government of Kenya and other countries assess future investments in the introduction and scale-up of the PrEP ring.

## Data Availability

The datasets used and/or analyzed during the current study available from the corresponding author on reasonable request.

## Abbreviations

AGYW: Adolescent girls and young women
ARV: Antiretroviral
ASPIRE: A Study to Prevent Infection with a Ring for Extended Use
DARAJA: Developing Risk Awareness towards Joint Action
DICE: Drop-in center
DREAMS: Determined, Resilient, Empowered, AIDS-free, Mentored, and Safe
FP: Family planning
FSW: Female sex worker
HOPE: HIV Open-Label Prevention Extension
IPM: International Partnership for Microbicides
MOH: Ministry of Health
MTN: Microbicide Trials Network
NASCOP: National AIDS and STI Control Programme
PEPFAR: U.S. President’s Emergency Plan for AIDS Relief
PrEP: Pre-exposure prophylaxis
PROMISE: Preparing for Ring Opportunities through Market Introduction Support and Knowledge Exchange
STEPS: Support Towards Expanded Prevention Services
UNAIDS: Joint United Nations Programme on HIV/AIDS
USAID: U.S. Agency for International Development
VMMC: Voluntary medical male circumcision
